# Barriers to postpartum diabetes screening: a qualitative synthesis of clinicians’ views

**DOI:** 10.3399/BJGP.2020.0928

**Published:** 2021-05-05

**Authors:** Georgina E Lithgow, Jasper Rossi, Simon J Griffin, Juliet A Usher-Smith, Rebecca A Dennison

**Affiliations:** School of Clinical Medicine, University of Cambridge, Cambridge;; School of Clinical Medicine, University of Cambridge, Cambridge;; MRC Epidemiology Unit, Institute of Metabolic Science, School of Clinical Medicine, University of Cambridge, Cambridge;; Primary Care Unit, Department of Public Health and Primary Care, School of Clinical Medicine, University of Cambridge, Cambridge.; Primary Care Unit, Department of Public Health and Primary Care, School of Clinical Medicine, University of Cambridge, Cambridge.

**Keywords:** diabetes, gestational, diabetes mellitus, type 2, postnatal care, postpartum period, qualitative research, systematic review

## Abstract

**Background:**

Gestational diabetes mellitus (GDM) is an important risk factor for developing type 2 diabetes mellitus (T2DM) later in life. Postpartum screening provides an opportunity for early detection and management of T2DM, but uptake is poor.

**Aim:**

To explore barriers to screening from clinicians’ perspectives to guide future interventions to increase uptake of postpartum screening.

**Design and setting:**

Systematic review and qualitative synthesis.

**Method:**

Qualitative studies included in a previous review were assessed, and then five electronic databases were searched from January 2013 to May 2019 for qualitative studies reporting clinicians’ perspectives on postpartum glucose screening after GDM. Study quality was assessed against the Critical Appraisal Skills Programmes checklist. Qualitative data from the studies were analysed using thematic synthesis.

**Results:**

Nine studies were included, containing views from 187 clinicians from both community and hospital care. Three main themes were identified: difficulties in handover between primary and secondary care (ambiguous roles and communication difficulties); short-term focus in clinical consultations (underplaying risk so as not to overwhelm patients and competing priorities); and patient-centric barriers such as time pressures.

**Conclusion:**

Barriers to diabetes screening were identified at both system and individual levels. At the system level, clarification of responsibility for testing among healthcare professionals and better systems for recall are needed. These could be achieved through registers, improved clinical protocols, and automatic flagging and prompts within electronic medical records. At the individual level, clinicians should be supported to prioritise the importance of screening within consultations and better educational resources made available for women. Making it more convenient for women to attend may also facilitate screening.

## INTRODUCTION

The metabolic stress caused by pregnancy can reveal a predisposition to health problems, and accordingly, gestational diabetes mellitus (GDM) is an important risk factor for the later development of type 2 diabetes mellitus (T2DM).^1^

GDM, defined as any degree of glucose intolerance first recognised during pregnancy,^2^ occurs in 5%–13% of pregnancies. The prevalence is increasing globally owing to the rise in risk factors like obesity and physical inactivity.^3^ Although hyperglycaemia typically resolves after delivery, women with GDM are approximately eight times more likely to develop T2DM than unaffected women, and one-third of women with GDM have been diagnosed with T2DM by 15 years postpartum.^4^

T2DM causes complications such as cardiovascular disease, nephropathy, and neuropathy that place a considerable burden on patients, health systems, and wider society.^5^ Women with a history of GDM represent a high-risk population identified at an early stage when they are eligible for risk reduction strategies and screening.^6^^,^^7^

Early detection of T2DM via screening enables early intervention, which reduces exposure to hyperglycaemia, and, therefore, risks of complications and premature mortality.

National and international guidelines recommend that women with GDM are tested between 6–13 weeks postpartum to exclude persisting diabetes, and then regularly screened for early hyperglycaemia after that, but specific tests and schedules vary between countries.^6^^,^^7^

The current UK guidelines from the National Institute for Health and Care Excellence (NICE) recommend *‘a fasting plasma glucose (FPG) test 6–13 weeks after the birth to exclude diabetes’* with the option of an FPG or glycated haemoglobin (HbA1c) test after 13 weeks if that was not possible.^7^ It is not specified whether primary or secondary care is responsible for conducting this initial test and, with uncertainty among clinicians, practice varies.^8^^,^^9^ If the patient’s test is negative, annual HbA1c testing should take place in general practice.^7^

Despite the guidelines and evidence supporting the effectiveness of early detection of T2DM, uptake of postpartum screening is poor.^6^^,^^10^ Often less than half of eligible women receive the recommended screening, with especially low rates in the groups most at risk, such as those from ethnic minorities and lower socioeconomic backgrounds.^11^^,^^12^

Furthermore, the percentage attending decreases year-on-year after delivery: one UK-based study reported that only 58% of women who have had GDM attended diabetes screening in the first year postpartum, and *<*40% attended in the second and third years.^13^ Two smaller, local studies found even lower annual rates of 18% after 3 years postpartum, and 20% over a 5-year period.^14^^,^^15^

**Table:** How this fits in

Postpartum diabetes screening after a gestational-diabetes-affected pregnancy is poorly attended, and barriers to attendance have been examined from women’s perspectives and in quantitative-based reviews from clinicians’ perspectives. This systematic review and thematic synthesis of qualitative studies included a wide variety of clinicians from different settings to provide deeper insight into handover difficulties between primary and secondary care as well as patient-centric barriers. It also revealed how a focus on short-term medical issues negatively impacts screening rates. Addressing these barriers through improved clinical protocols, better reminder systems, and more convenient testing options may allow for improved uptake of screening.

Understanding the reasons for low screening uptake is essential for developing effective strategies to improve screening rates. A recent systematic review identified a number of influences on postpartum screening from the perspective of women who had had GDM.^16^ This found that the manner in which their health care was provided affected their motivation to attend screening. For example, clinicians discussing postpartum screening throughout pregnancy could help women to prioritise it, whereas not receiving an invitation for the test could imply that attendance was not important.

The level of concern that women had regarding developing diabetes also influenced screening motivation, and the logistics of testing within a busy postpartum schedule could make attendance difficult. Therefore, understanding attitudes towards screening and the barriers from the perspective of clinicians is important. A previous systematic review reported clinicians’ perspectives on this topic, but only included two qualitative studies.^17^

The aim of this review, therefore, was to provide an updated and expanded qualitative synthesis of clinicians’ perspectives on the issue in order to better understand the challenges faced in current practice and to inform strategies to increase attendance at screening.

## METHOD

### Search strategy

The authors assessed the two qualitative studies included in the earlier review by Van Ryswyk and others^17^ that searched the literature from database inception to 2013. The literature was then searched from January 2013 up to May 2019 in MEDLINE, Embase, PsycINFO, CINAHL, and the Cochrane Library electronic databases.

The search strategy (see Supplementary Table S1 for details) was informed by the previous review,^17^ and it was ensured that key, recent studies already known to the authors were identified. Reference lists of included studies were screened for citations not identified by this search.

### Study selection

Peer-reviewed articles that examined clinicians’ (defined as any healthcare professional involved in the pre- or postpartum care of those with GDM) perspectives on postpartum glucose screening were included. Both qualitative and mixed-methods studies were eligible. The search was restricted to English-language articles — the language of the authors — because it was considered particularly important to have a thorough grasp of the language for a qualitative analysis where meaning is central, and the study did not have the resources to have the manuscripts translated in this instance.

After deduplication, all titles and abstracts were assessed against the selection criteria by two authors. Both authors reviewed about 10% of the citations to ensure agreement. Any differences were discussed, and the selection criteria were refined and elaborated in conjunction with the other authors so that they could be applied consistently. Full–text articles were then acquired and rechecked against these criteria. Study characteristics were extracted into an Excel spreadsheet and crosschecked.

### Quality assessment

The quality of each study’s qualitative research was assessed against the Critical Appraisal Skills Programmes (CASP) checklist for qualitative research.^18^

Each checklist was reviewed and any differences were discussed. Scores of 0, 0.5, and 1 were awarded for answering ‘no’, ‘unclear’, and ‘yes’, respectively, to each of the 10 questions. No studies were excluded based on quality.

### Thematic synthesis

A thematic synthesis was conducted, as described by Thomas and Harden,^19^ using NVivo (version 12). This approach was chosen above other strategies for qualitative synthesis in order to enable the authors to develop novel or deeper understanding of the phenomena reported in the primary studies using an inductive approach. The data analysed came from the text produced from qualitative methods under the heading ‘Results’ in included studies.

From these, a coding frame was formed, which was used to develop descriptive themes. The initial coding frame was developed by focusing on three studies. Two authors met to agree the codes, and then applied them to the other studies independently, adding new codes as they came up and comparing the results. Descriptive and then analytical themes were developed through a collaborative process between authors. Each code was summarised and similar ideas were grouped to describe the concepts presented in the studies. The two researchers then went beyond these descriptive concepts to explore analytical themes. This iterative process involved revising the descriptive groups and exploring how they fit together to synthesise the findings into a cohesive set of themes that directly addressed the concerns of the review. Researchers worked independently and then together, as well as with the wider research team to further refine the analytical themes.

## RESULTS

**Figure 1.**
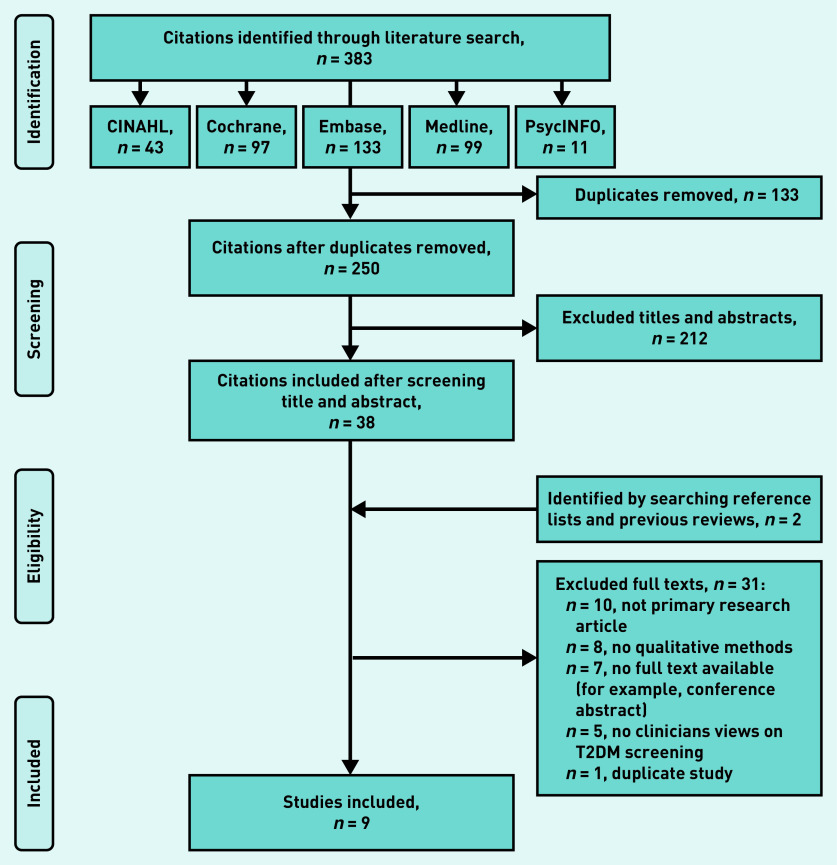
*PRISMA diagram. T2DM = type 2 diabetes mellitus.*

**Figure 2.**
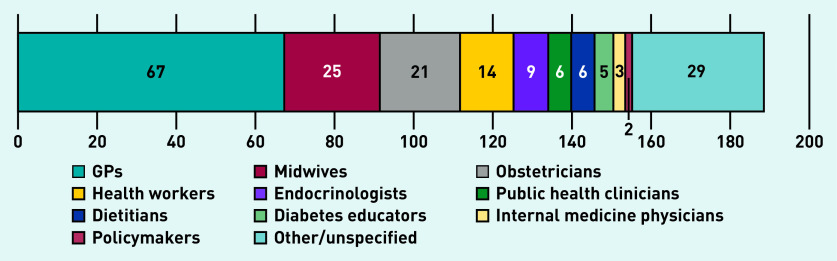
*Types of clinicians represented in the qualitative synthesis.*

Nine studies were included after screening 250 citations and reviewing 38 full texts (Figure 1). For the characteristics of those studies, see Supplementary Table S2. The median number of participants was 25 (range 10–44), with 187 clinicians in total. The most commonly represented clinicians were GPs (*n* = 67), then midwives (*n* = 25), and then obstetricians (*n* = 21), but a wide variety made up the rest, including 29 ‘other/unspecified’ (Figure 2). Participants came from hospitals (*n* = 2/9), community-based services (*n* = 2/9), or both (*n* = 5/9). The majority of studies used interviews — either face-to-face or telephone — and nearly all studies were set in high-income countries (Australia [ *n* = 4], Singapore [ *n* = 1], and the US [ *n* = 2]). Most of the studies were found to be of good quality (mean CASP score = 8/10), as detailed in Box 1. The most common weaknesses were lack of sufficient consideration of both the relationship between the researcher(s) and participants, and ethical issues (the studies had been approved by ethics committees but the authors did not report any details about how ethical standards were maintained).

**Box 1.** Quality assessment of the studies included in the qualitative synthesis

**CASP appraisal criteria**	**Doran, 2010^24^**	**Wilkinson, 2014^27^**	**Bernstein, 2016^22^**	**Campbell, 2017^25^**	**Pennington, 2017^28^**	**Hewage, 2018^21^**	**Muhwava, 2018^23^**	**Kilgour, 2019^26^**	**McCloskey, 2019^20^**
**Clear aims?**	✓	✓	✓	✓	✓	✓	✓	✓	✓
**Qualitative methodology appropriate?**	✓	✓	✓	✓	✓	✓	✓	✓	✓
**Appropriate research design?**	✓	✓	✓	✓	✓	✓	✓	✓	✓
**Appropriate recruitment strategy?**	✓	✓	**?**	✓	✓	✓	✓	✓	✓
**Appropriate data collection?**	✓	✓	✓	✓	✓	✓	✓	✓	✓
**Adequate consideration of relationship between researcher and participants?**	**?**	**X**	**X**	**?**	**X**	**X**	**X**	**X**	**X**
**Consideration of ethical issues?**	**?**	**?**	**?**	**?**	**?**	**?**	✓	✓	**?**
**Data analysis sufficiently rigorous?**	X	**?**	✓	✓	✓	**?**	✓	✓	✓
**Clear statement of findings?**	**?**	✓	✓	✓	✓	✓	✓	✓	✓
**Valuable to the literature review?**	**?**	**X**	✓	**?**	✓	✓	✓	✓	✓
**Total score**	7	7	8	8.5	8.5	8	9	9	8.5

✓ = *Clearly met criterion.* ? = *Unclear if criterion met.* X = *Criterion not met. CASP* = *Critical Appraisal Skills Programme.*

Three main themes were identified:

- difficulties in handover between primary and secondary care (ambiguous roles and communication difficulties caused by poor-quality information or technological barriers);
- the short-term focus in clinical consultations (clinicians underplay risk so not to overwhelm patients and have competing priorities in postpartum consultations); and
- patient barriers (difficult environment and concerns about the test).

These are explained further on and summarised in Box 2. Although it was not generally explicit whether these barriers were relevant only to the first screening test or across subsequent visits, most barriers are likely to apply across the timeframe.

**Box 2.** Overview of the findings of the qualitative synthesis

**Theme**	**Quotes/explanation**
**1. Difficulties in handover between primary and secondary care**	**Ambiguous roles and responsibilities** *‘Look how confusing it is. Who do you see, when do you see them, who do you refer them to? There’s no simple pathway.’* (health professional)^25^ *‘One of the core issues is that you’ve got a resource constrained situation,* [clinicians] *are full to the brim* […] *Their job is 120%, so anything else you give them, is a problem.’* (public health specialist)^23^ *‘It’s not that people are not aware that* [screening] *needs to be done, it is because the environment will be challenging for people to be doing OGTTs. That’s associated with human as well as financial resources.’* (obstetrician)^23^ **Communication difficulties** *‘I personally hate those* […] *discharges, they are impossible! I mean something much more succinct, a summary. They obviously have printed the entire record!’* (GP)^26^ Discharge summaries that: ‘*Tell us how she is going, what you have done, what you are going to do and what you want me to do.’* (GP)^26^ *‘No proper system to identify and retain patients in the current care model.’* (primary care clinician)^21^
**2. Short-term focus in consultations**	*‘We present it like “okay, you have GDM, it’s a potential risk, but it’s not technically affected the baby per se.” I think we may be part of it* […] *that we maybe simplify it so they don’t see it as “oh it’s not going to kill my baby right”.*’ (community nurse midwife)^20^ *‘Our focus is the pregnancy, keep the sugar down, try and have a healthy baby and a mother that’s not injured during the birth. And we don’t think too much to the afterwards.’* (professor in obstetrics)^23^ *‘You see the patient and talk about the baby but not beyond.’* (unspecified)^22^
**3. Patient barriers**	*‘They’ve got too many other things.’* (GP)^28^ *‘They’re so focused on the here and now they can’t even comprehend what might happen in the future.’* (health worker)^25^ *‘The drink, a lot of people don’t like it, so they won’t come in for it. And the time it takes to have it as well.’* (health worker)^25^

*GDM = gestational diabetes mellitus. OGTT = oral glucose tolerance test.*

Variation was also identified in the roles and priorities of practitioners in different settings. As explained throughout the text and summarised in Box 3, hospital clinicians tended to focus on management of GDM during pregnancy, while GPs were concerned with postpartum follow-up and the lack of information or resources to facilitate it.

**Box 3.** Variation between hospital and community practitioners

**Clinician**	**Their role/priorities**	**Consequences for postpartum screening**	**Quotes**
Hospital clinicians	GDM management main priority and prevention much lower priority Role ends at ∼6 weeks postpartum, so no control over follow-up	Viewed as important but not their role Long-term risk communication is lacking: Fear of overwhelming patient and impacting GDM control No time for discussions	*‘Our focus is the pregnancy, keep the sugar down, try and have a healthy baby and a mother that’s not injured during the birth. And we don’t think too much to the afterwards.’* (professor in obstetrics)^23^ *‘I don’t feel equipped to handle a positive test, so I think that’s why I don’t have them follow-up with me* […] *I want them to follow-up with their primary care provider, so they can say “Okay, this is how we’re going to go forward in the future. And I feel like I’m not equipped to give them that information, so it wheels down to be like “Test is normal, you know, bye”*.’ (community nurse midwife)^22^
Primary care providers	Primary prevention a main priority but often lack sufficient information to support this process	Viewed as important, and their role, but the barriers prevent them achieving it	*‘It is the GP role, our domain to follow that up.’* (GP)^26^ *‘I would like to know what I am meant to do to be fairly clearly guided as to what to check when or what to do next time, or when to be seen back again.’* (GP)^26^

*GDM = gestational diabetes mellitus.*

### Difficulties in handover between primary and secondary care

#### Ambiguous roles and responsibilities

Ambiguous roles in GDM follow-up meant that it was not done systematically and so patients fell through the gaps during handover. It was not clear whose responsibility it was to order or conduct screening tests, nor who was expected to communicate the results to the patient. The absence of standardisation left the handover process haphazard and made it easy for patients to be lost to followup.^20^ Singaporean participants contrasted their system to the Australian one, where a national registry of patients facilitated systematic follow-up.^21^

The timing of the test exacerbated those challenges; patients were discharged from obstetric care at 6–8 weeks and so the postpartum test *‘fall* [s] *into a gap of the shift to primary care’* in *‘the chasm between specialties.’* (community nurse midwife).^22^ If hospital clinicians ordered the test at the 6-week visit they did not receive the results until after the patient had been discharged from their care, and if they advised their patients to have testing they had no way of checking if it had taken place.^23^ Ordinarily, hospital clinicians did not learn what happened to their patients unless they became pregnant again.^20^

Generally, screening was felt to fit better within primary care where there was better continuity of care.^21^ However, the postpartum period left little time for the transition to community care to occur, and it often took longer than 13 weeks before Australian primary care clinicians saw the patient.^20^ Furthermore, their clinician needed to know of their history of GDM and their responsibility to perform a screening test. This required good-quality communication from the hospital, as described in the second barrier to screening in this theme.

Finally, lack of time and resources hampered both the motivation and the ability of hospital and community clinicians.^20^ Clinicians whose jobs are already *‘120%.’* (community nurse midwife).^20^ were unlikely to voluntarily take on extra duties administering screening tests.^23^ Even though many clinicians were aware of the need for screening, a heavy patient load and already limited time per patient created a challenging environment for testing.^21^^,^^23^ Furthermore, some health services lacked physical resources, with centres either not set up for testing or lacking capacity or funding to offer it.^23^^–^^25^

#### Communication difficulties

Poor-quality communication that lacked information relevant to later care and technological barriers (including a lack of integration between services and red flags) both contributed to primary care clinicians not knowing of a patient’s GDM diagnosis and need for postpartum screening.

Communication between hospital and community care services was *‘hit and miss’* (GP).^26^ Referral letters and discharge summaries often did not *‘get completed very well.’* (professor in obstetrics).^23^ Clinicians were frustrated at the lack of discharge protocols,^20^ which meant that discharge summaries were completed with little guidance and according to individuals’ inclinations. GPs said that the summaries they received often lacked clear guidance for follow-up, which left them inadequately informed.^26^

The inclusion of too much irrelevant information also made salient points difficult to find by primary care physicians, who were already pressed for time. GPs wanted a summary: *‘a punchy paragraph letter saying these were the issues, this is the follow-up from the GP, what is required’* (GP).^26^ Instead, they often received a *‘generic printout of all care provided throughout the course of the hospital admission’* (GP),^26^ which meant that the patient’s GDM status may go unnoticed and overlooked, which negatively influenced completion of care.

A lack of integration of medical records meant that hospital records often could not be accessed by the community and vice versa. Any results of investigations and diagnoses could not be accessed unless included in letters, which may not even reach the primary healthcare services.^22^^,^^23^ There was a general lack of communication channels within the health service.^23^ Even within specialities there was often a lack of efficient referral and tracking systems, and between hospital and primary care it was largely absent.^22^ There was a need for improved, integrated electronic medical records that could be easily accessed by both hospital and community, and for items like the need for screening tests to be readily seen.^21^

A further technological issue was the lack of effective reminder *‘red flags’* alerts to prompt testing in primary care.^25^ Without such alerts, unless the GDM diagnosis was made obvious in the notes (for example, in the problem list) it was unlikely to be seen by the new clinician, leaving them unaware of the diagnosis and subsequent need for screening.^22^

The electronic reminder system within primary care was also an issue: GPs *‘had to remember to click “reminders” in the electronic medical record system’* and the fact that was not automatic made it *‘easy to miss’* (GP).^27^ All of these contributed to the patient’s need for GDM screening being overlooked.

### Short-term focus in consultations

Difficulties also arose both during and after pregnancy in the individual consultations, which tended to focus on the short-term challenges of GDM or having a new baby present. A tendency to underplay future dangers and the distraction of competing priorities meant that T2DM risk was not a major feature of discussions, and, therefore, often not a notable part of women’s concerns.

When communicating a GDM diagnosis, clinicians in the hospital were apt to focus on the immediate challenges it presented and were reluctant to emphasise women’s future T2DM risk for fear of overwhelming them. This could lead to the patients misunderstanding GDM as an exclusively short-term challenge. Clinicians wanted to present the diagnosis in a way that motivated the patient to control their GDM during pregnancy to protect the infant, rather than frightening them and impairing their ability to do so.^20^^,^^21^ Pregnancy was already a vulnerable time, and so this was a *‘balancing act’* (community nurse midwife) between reassurance and risk communication.^20^ As a result, clinicians often opted for a short-term vision of working towards a healthy baby and did not go in-depth into future implications for the women themselves.^22^ This meant that patients often viewed the end of the postpartum period as an end to the complications of pregnancy, and left obstetric care without understanding that they were at significantly increased risk for T2DM; and, consequently, the importance of being screened.^20^^,^^22^

Once back in primary care, consultations again focused on the more urgent matters. Breastfeeding, infection, and caesareansection healing all took precedence over conversations about glucose screening.^20^ Typically, there was a single consultation for both mother and baby. Visits become *‘baby, baby, baby’ (*GP),^26^ and their development, presenting symptoms, or vaccination requests crowded out the women’s care as clinicians tried to fit everything in to an already time-stretched visit.^27^ In the limited time and in the midst of all the different guidelines, agendas, concerns, and recommendations, postpartum screening could get lost.^26^

### Patient barriers

Finally, many clinicians gave their perspective on what they perceived to be the barriers in the women’s lives that prevented them from attending screening.

Competing priorities were perceived to be the major barrier to women attending. The postpartum period was busy, with many demands and new responsibilities to be juggled.^20^^,^^28^ In this time, screening had a *‘lack of saliency’* (primary care clinician)^21^ as *‘they’re so focused on the here and now’* (health professional).^25^

With their own health often a low priority,^28^ screening may simply have been forgotten amid other worries.^26^ Furthermore, screening was time consuming and conflicted with childcare,^28^ and the systemic divide between maternal and child healthcare services was a further inconvenience. What is more, early T2DM does not typically present with a lot of symptoms: it is not painful and the mother feels well, which provided little motivation to attend screening.^22^^,^^23^ Amid the busyness, with no immediate consequences for not attending,^20^^,^^25^ screening was often not a priority.

A variety of other factors created an environment that made it difficult to be screened: financial difficulties, a lack of autonomy in choices, a peer group that was unaware of the risks, and lack of a primary care provider all contributed.^21^^,^^23^^,^^28^ Moreover, it was those most at risk who most frequently faced these struggles.^25^ Difficulties also arose from the test itself; it could be unpleasant to take,^25^^,^^28^ and stigma around a diabetes diagnosis means that women *‘want to avoid* […] *that shame’* (health professional)^25^ and criticism.

## DISCUSSION

### Summary

This synthesis of qualitative studies has for the first time allowed an in-depth exploration of the barriers to post-GDM T2DM screening from the perspective of 187 clinicians from a wide range of healthcare settings. Handover difficulties, poor information transfer, and reluctance to communicate risk were found to add to the obstacles that patients encountered that contribute to poor attendance. Addressing these barriers by methods such as local or national registers, improved protocols, and reminder systems for both women and clinicians has the potential to increase rates of screening, and consequently, reduce morbidity from T2DM. Making it more convenient for women to attend may also facilitate attendance.

### Strengths and limitations

A major strength of this analysis was the inclusion of a wide range of different healthcare professionals (Figure 2), providing multicultural perspectives across the spectrum of prenatal, postpartum, and primary care, from a range of public and private health settings from university hospitals to village health centres. This allowed the analysis to distinguish between hospital and primary care clinicians; their different priorities, barriers, and facilities for screening.

Nevertheless, all of the studies were in English, with the majority (*n* = 7/9) set in high-income countries and Australia (*n* = 4/7). Identification of country-specific issues may limit the applicability of results to the UK; for example, GPs in Australia may not always see their patients before 13 weeks postpartum but the 6-week mother and baby health check is well-attended in the UK.^20^ However, others are likely to be highly relevant to other countries, and surveys in the UK have indicated that there is still confusion and disagreement about responsibility for testing among clinicians.^8^^,^^9^ Although some aspects were more pronounced in the studies from lower-income countries, both drew similar conclusions despite the varied settings.

The studies included in this synthesis were of good quality as assessed by the CASP tool, with a mean score of 8/10. Their aims, research design, recruitment strategy, data collection and analysis, and findings were generally well described. However, it was usually unclear to what extent ethical considerations were taken into account, as was the relationship between researchers and participants. In addition, recruitment methods relied on either purposive or convenience sampling, which could contribute to sampling bias and limit external validity of findings. Although the timing of the screening test considered was often unclear, studies across a definitively longer timeframe may reveal barriers that were not identified in this review. Furthermore, the patient barriers were limited to what could be externally perceived by clinicians, although these results do align with those from a review from the women’s perspective.^16^

### Comparison with existing literature

The difficulties in handover between primary and secondary care, and the patient-centric barriers surrounding the test environment were identified previously in Van Ryswyk *et al*’s 2014 systematic review.^17^ Focusing exclusively on qualitative studies enabled this study to explore in greater depth those barriers raised by the primarily quantitative/survey-based review. For example, Van Ryswyk *et al* noted that *‘more research is required into developing strategies* […] *such as ways to improve communication between clinicians regarding GDM diagnosis and care.’* ^17^ It was possible to determine more precisely the ways in which communication was deficient (such as the absence of GDM status in discharge summaries), and so, there is potential to address it more directly and effectively. Additionally, a novel theme was identified whereby focus on short-term challenges led to inadequate communication of risk in consultations.

The importance of this lack of communication is seen in studies of women with a history of GDM. Dennison *et al* found that the behaviour of clinicians could either conflict with or reinforce prioritisation of screening.^16^ Emphasising postpartum risk and follow-up increased patient motivation to attend, while prioritising the baby led to less information on maternal risk and less motivation. There was additional agreement between the patient-centric barriers identified by mothers and those identified by clinicians in this review, particularly the competing priorities and dislike of the test itself. Although mothers were less aware of system-level handover challenges, they also noted that GPs often lacked knowledge of their GDM diagnosis.

The importance of a good handover and communication of patient information between primary and secondary care is not unique to GDM follow-up. Absence of a standardised discharge protocol, fragmented communication, and lack of information on plans for follow-up affects patients’ experiences and health outcomes across a wide range of conditions.^29^^–^^31^ Therefore, addressing communication deficits is important in a variety of specialities.

One study of clinicians’ views of long-term complications of GDM was published after completing the literature search. Nagraj *et al*’s^32^ study had a rural Indian setting with greater social and gender inequality. Competing priorities, vague roles, and resource limitations were similar to the findings of the present study; however, they also found that many physicians lacked awareness of the long-term implications of GDM, which was not prominent in the results of the present study. In some settings, education of physicians needs to be a greater priority.

### Implications for research and practice

This study found that handover difficulties, poor information transfer, reluctance to communicate risk, and obstacles to patients’ attendance contribute to poor attendance. Here are suggested approaches to overcome these. These should be considered in the context of each healthcare setting.

There is a need to make the process of handover to primary care more systematic. One way this could be brought about is through the creation of national strategies that clarify responsibility in GDM follow-up in countries that do not currently have a system in place. This would involve improved clinical protocols and referral pathways; methods that have been associated with higher rates of postpartum screening.^33^^,^^34^ The ability to identify and retain patients would assist in this endeavour, such as by creation of local or national registers of women who have had GDM. This may aid systematic follow-up across different locations and time points because it could act as a common resource for all clinicians and remove the challenge of transferring information between institutions. Such approaches have significant potential to improve testing rates.^35^ The Saudi National Diabetes Registry and the Australian National Gestational Diabetes Register are examples,^36^^,^^37^ although more research is needed to evaluate how such registers can be optimised for GDM followup, as these countries still have a way to go with screening attendance. For example, the Australian studies included in the present research were all performed after the register’s inception in 2011. Registers already facilitate other aspects of diabetes care in the UK, such as the national register for diabetic retinopathy screening and the local GP registers that cover annual diabetic reviews.

Another approach to better facilitate handover is revamping discharge summaries and reminder systems to make the diagnosis and need for screening more obvious in the records. Improvements to discharge summaries may include updating templates to prioritise GDM status and follow-up plans, the inclusion of automated orders for postpartum testing on discharge, and raising awareness of the importance of documenting GDM. There is some evidence that these approaches increase testing rates.^38^ Improvements in reminder systems include prompts for clinicians to ask about history of GDM, and flags in the electronic medical record for a GDM diagnosis and need for screening. These have previously shown some success for GDM^33^^,^^39^ and other conditions.^40^

To address the poor communication of T2DM risk to patients, a training intervention may be used to improve knowledge surrounding GDM among the workforce. This would aim to promote consistent messages across services, encourage clinicians to prioritise T2DM discussions, and alert women with GDM of the need to book an extended appointment time for postnatal checks (information that could also be communicated by those responsible for making bookings at the GP clinics). Such training interventions have been shown to be successful in modifying other clinical behaviours;^41^^,^^42^ however, risk awareness does not always translate to increased attendance at screening, so communication needs to be optimised.^16^ Alternatively, more resource-intensive approaches include addition of a separate visit exclusively devoted to education on postpartum T2DM risk or the creation of a new category of outreach workers who are designated providers for diabetes care.

To address the barriers for women explored in this review and reported by women with GDM themselves,^16^ there is a need for better information and educational resources, integration of visits, more flexible options for test taking, and reminders. Educational resources would provide written information at a fitting level and in multiple languages. It may also be provided by indigenous health workers or mass media public health campaigns. Integrating visits could combine the test with other appointments — such as the child immunisation programme or cervical cancer screening that are available in the UK and many other countries — to increase convenience, but does raise concerns about overloading staff.

Other more flexible options for test taking include early testing at the hospital, having drop-in locations open at more convenient times, and the opportunity to take the sugar drink or the entire test at home. Reminders by post, email, or telephone may also improve testing.^43^^,^^44^

All these strategies need to be evaluated for feasibility, acceptability, and effectiveness, and the implications on resources should be considered; particularly when implementing them in countries outside of those represented in this review. Involving clinicians in developing and evaluating these strategies will be key.
